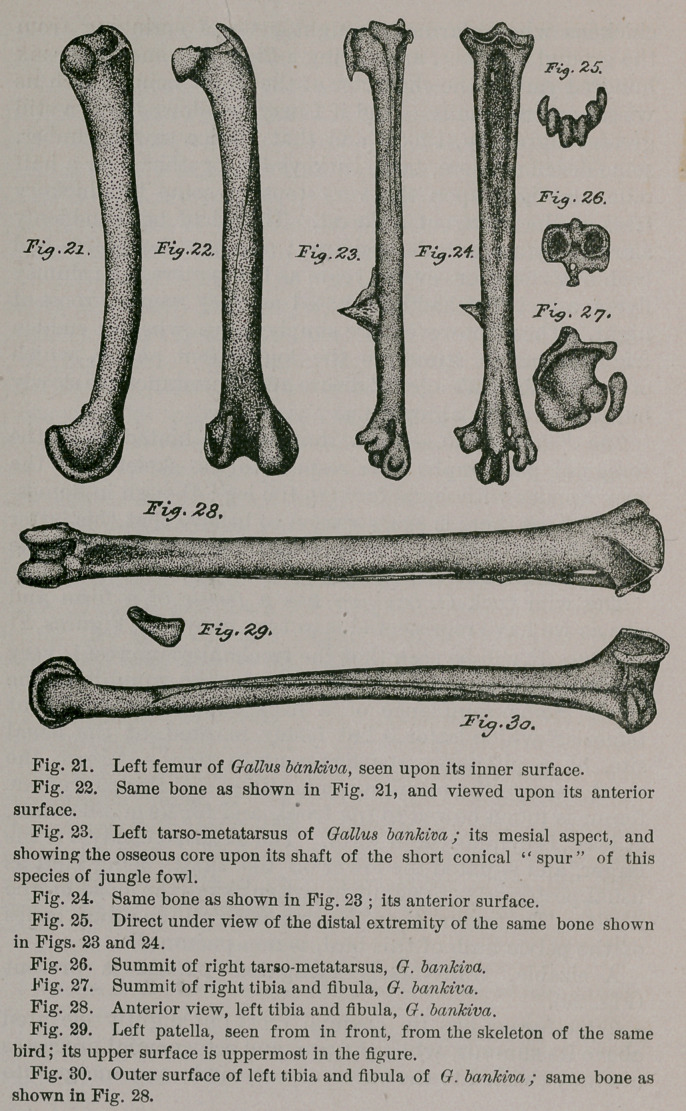# Observations upon the Morphology of Gallus Bankiva of India

**Published:** 1888-10

**Authors:** R. W. Shufeldt


THE JOURNAL

OF

COMPARATIVE MEDICINE AND SURGERY.

VOL. IX. OCTOBER, 1888. No. 4

### ORIGINAL COMMUNICATIONS.

### ART. XXI.—OBSERVATIONS UPON THE MORPHOLOGY OF GALLUS BANKIVA OF INDIA.

(INCLUDING A COMPLETE ACCOUNT OF ITS SKELETON.)

BY R. W. SHUFELDT, M.D., C. M. Z. S.

In the *Ibis* of 1881 (pp. 174–77), Forbes makes an appeal to ornithologists, in various parts of the world, to send him material in order that he may complete some of the work on the Anatomy of Birds, left by Garrod; and the last paragraph of this paper reads as follows, and he says: "I may add that I shall be very grateful if some of the many ornithologists resident in India would procure me about half a dozen specimens (adult) of wild-shot *Gallus bankiva*, in spirits, or even skeletons. As the first part of Prof. Garrod's treatise is devoted to the anatomy of the Fowl, it is desirable to have wild specimens of it for dissection, or at least to describe the bones from them, and not from any of our domestic races." We now know that Forbes met with his death at Shonga, Africa, before he was enabled to complete any such piece of work as he here points out; a piece of work, I may add, that no one will gainsay the necessity for, owing to the fact that the fowl has been utilized as sort of a standard of the bird skeleton in text-books, works on embryology and morphology, all over the world, and, yet, so far as the writer knows, the type skeleton of the species has not been fully treated of, while, as we all know, the greatest amount of variation is now to be found in the skeletons of the domestic species.

Huxley, too, has alluded to the desirability of having a complete description of the skeleton of the wild-shot *Gallus bankiva*, of India, and Darwin, in his "Plants and Animals under Domestication," very fully speaks of the expediency of possessing such a treatise, and points out, with great clearness, the wide differences that may exist in the skeletons of various forms of fowls.

With these facts before me, I some time ago placed the matter before Dr. Conklin, who at once interested himself in it, and he wrote to Dr. Richard W. Burke, of Cawnpore, India, for specimens, and thanks to these gentlemen, I have this day received from Jubbulpore, India, alcoholic specimens of a pair of wild-shot, adult *Gallus bankiva*, a cock and hen, so that, in the present memoir, I am enabled to offer an account of the anatomy of these birds, but, more particularly, a full description of the skeleton.

The pair was in good condition upon coming into my hands, although a few bones had been broken by the shot, and the hen had been eviscerated before shipping. As the plumage of these birds is well known, I will pass no remarks upon it here, though I may say that I find ten remiges in the wing and fourteen rectrices in the tail of either specimen; and the cock appeared to be about one-fourth larger than the hen, and the two middle feathers of his tail are somewhat elongated, which is not the case in the latter. A very diminutive lobe evidences the presence of a comb on the head of the hen, while both comb and wattles are of very small size even in the male bird; and in him only is an inconspicuous conical spur, to be found on either tarsus at the most usual site.*

In general form they agree quite closely with small specimens of the best examples of our domestic game fowls, and, upon carefully plucking the cock bird, I find its pterylosis corresponds, in all essential particulars, with the pterylosis of *G. bankiva*, as drawn for us by Nitzsch, although I must believe, from the form of the comb and wattles, in this eminent anatomist's figures, he had before him a specimen of the barn-yard fowl.†

A circlet of dense, fine feathers, completely surround the aural aperture, and the uropygial papilla is tufted. In both sexes, the alula supports *four* flight feathers, the outer one being much the longest, and the others graduated to a very small inner one. Cutting down upon the carotids, I find them double, as already stated to be the case by Garrod; and further, I am enabled to verify that talented anatomist's account of the form of the trachea and syrinx in *Gallus bankiva*, for I carefully removed it in the cock bird, and compared it with his excellent figures.‡

Presenting nothing especially noteworthy, the average-sized, median and single crop, upon being opened was found to contain several varieties of small seeds, numerous beetles, pupæ, buds, and two or three land shells (*Helix ?*). The lower opening of this crop is in contact with the syrinx and both of these structures are deep situated in the chest.

---

*In this connection, I am permitted to quote from a very interesting letter written by Dr. Burke to Dr. Conklin, dated Jubbulpore, India, 14 March, 1888, wherein the former says that "the term 'wild fowl' in India and England, is applied to birds of the *Anseridæ*, *Tadominæ* and *Anatinæ* families, and *not* the jungle fowl as believed by some persons. In my opinion, the original stock of the domestic fowl comes from the jungle fowl, as jungle fowl can be so easily tamed, especially when the eggs have been hatched under a domestic hen. The jungle fowl will breed in captivity, and we know of a hybrid between it and the *Gallophasis albocristatus*, having the head, breast and back of the pheasant and tail of the jungle cock, with red feathers and no crest."

R. W. S.

† "Pterylography." (Eng. trans., 1867) Taf. VII., Figs 5 and 6.

‡ Garrod, A. H. Coll. Sci. Mem., Lond., 1881, pp. 174 and 501, Figs. 32, 33.

Passing next to an examination of the patagial muscles of the shoulder, I find a very small *tensor patagii longus*, coming off from the pectoral muscle and lying flat against the *t. p. brevis*, from which it also receives fibres. Its tendon has the usual course and insertion. Although the *temporo-alaris* is fairly well developed, the *dermo-tensor patagii* is only represented by a few fibres, while its tendon is lost in the integuments of the propatagium, which latter here is barely manifest.*

Nearly as large as the biceps, the chief muscle of this group is the *tensor patagii brevis*, which, arising as usual from the os furcula and coracoid, covers, as it descends the dorsal aspect of the shoulder, and its broad, single, though slender tendon is inserted into the fascia only of the *extensor metacarpi radialis longus*. We also find present a small *tensor patagii accessorius*, arising well up on the fascia of the biceps muscle, and its well-defined cord-like tendon passing to merge with the tendon of the *t. p. longus*, at about the middle of its course.

This arrangement of these muscles seems to agree very well with the corresponding ones, as we find them in pigeons, although even here the forms vary to no little extent.†

One is struck at once in dissecting these wild fowls, by the extreme delicacy and whiteness of their flesh. All three of the pectoral muscles are present, and, as in other members of the group, the *pectoralis major* is of great extent. Upon dissecting out the muscles of the thigh, I find them to agree in all matters with the account left us of them by Garrod.‡

A very handsomely developed *ambiens* is seen; and the nervous, venous and arterial supply of this pelvic limb seems to depart in no way from a condition of affairs well known to obtain among the typical *Gallinæ*.

---

* SHUFELDT, R. W. "A Review of the Muscles used in the Classification of Birds" JOUR. COMP. MED. AND SURGERY, Vol. VIII., No. 4, 1887, pp. 321–344.

† PARKER, T. J. "Zootomy." Lond. 1884 (Chapter on the Pigeon), pp. 251, 252.

‡ *Ibid.* p. 188, Fig. 1.

Having the usual origin and insertion, a beautiful *expansor secundariorum* is found to be represented ; and should one care to study this curious little muscle, I know of no form in which it can be more successfully done than in the species before me.

Opening the thoracic and abdominal cavities, I note the presence of the fatty *great omentum,* spreading over the entire mass of abdominal viscera, a large, *triangular* obturator internus muscle, and the system of air-sacs far better developed than I ever found them in any of our domestic fowls; indeed, in some varieties of these latter, the air-sacs show evident signs of atrophy. The *intestinal cœca* are of unusual length, each measuring fully fifteen centimetres, and terminating in bulbous extremities. Unfortunately, the liver and other parts were so decomposed that I could do nothing with them. As to the gizzard and its special form, a better idea can be obtained from my drawing of it, herewith presented, than any written description can convey. (Fig. 1.)

Nothing worthy of special record characterizes the heart in this specimen.

With this brief description, then, of these various "soft parts," and other characteristics of this wild Indian fowl, we will now proceed to investigate the skeletons of the pair before us, which is really the main object I had in view in writing this paper. And first, as to the most interesting part of their osseous system, or, *the skull.*

Fig. 1. Part of intestinal tract, and the gizzard of the wild *Gallus bankiva* of India. Drawn life-size by the author from the specimen.

Darwin, when he came to compare the extraordinary forms the skull assumes in many of the domestic breeds of fowls with the skull of the wild *Gallus bankiva*, pointed out for us quite a number of the salient features in the skull of the latter species,* and it will be my aim in the present paper to discuss these more in detail, and without any attempt to make comparisons with domestic species, touch more fully upon the differences found in the skulls of the male and female *Gallus bankiva*, as seen in the two specimens now before me. In doing this, I must once more remind my reader of a fact that I have so often insisted upon in others of my memoirs, and that is, that the individual variation of the skull for the same species may be marked to a marvelous degree in some specimens, and we may have as an example a thick and unperforated interorbital septum in the skull of one bird, and a thin one, showing a large vacuity in the same osseous partition in the skull of another individual of the same species. Still more manifest differences may extend to size and even form of such parts as beak, brain-case, and basi-temporal area. So, then, under such circumstances, the description I here present for the skull of the jungle fowl will hardly hold good for all details in other specimens of the same species, although, no doubt, the main characteristics will be found descriptive of the vast majority of skulls. These remarks are equally applicable to the remaining parts of the skeleton.

As is generally the case with gallinaceous fowls, the *premaxillary* develops conspicuous nasal and maxillary processes ; the former being longitudinally separated for their hinder two-thirds, with the posterior ends almost entirely covering the ethmoid where it makes its appearance anteriorly between the frontals. In domestic fowls the ethmoid is sometimes not overlapped at all at this point, but is exposed as an escutcheon-shaped area of some considerable size.†

---

* DARWIN, CH. "Animals and Plants Under Domestication." Vol. I., pp. 315–321, Am. Ed., 1868.

† See Prof. W. K. PARKER's figure (Fig. 19) of the skull of common fowl in the 9th ed. of Brit. Encyclopædia, Vol. III., p. 709, *eth.*

The "maxillary processes" are thin and pointed, and extend posterior to the point of meeting of the distal end of the nasal, on either side. Between the narial apertures the premaxillary is very narrow, and the osseous culmen formed by this bone presents a double arch along its anterior two-thirds; one over the nostrils, and the other over the fore-part of the beak (Fig. 2). These two curvatures are best seen in the skull of the hen bird at my hand. Anteriorly the osseous superior mandible is rounded, while its lateral edges are sharply caltrate, and beneath, for its fore-part, it is much concaved, as in most *Gallinæ.* Either external narial aperture is very large, and of a subelliptical outline, though with the arc broader behind than it is in front; while no median, bony, internasal septum is developed between these openings. In the skull of my female specimen the naso-frontal sutures are completely obliterated, but they can be faintly traced in the skull of the cock (Fig. 3). On the other hand, the anterior processes of a *nasal* bone only partially anchylose with the premaxillary above and below, and with care these latter bones can be easily detached along their sutural lines. A nasal bone is separated from the fellow of the opposite side by the median, backward-extending process of the premaxillary, as is the case in most of the domestic species. However, Darwin calls attention to the fact that in the "Sultans" (a Turkish breed), "the inner processes of the nasal bones were ossified together."*

A *lacrymal* in *Gallus bankiva* is a small scale-like bone, sub-triangular in outline, and freely articulated along its inner border to the anterior naso-frontal margin of the orbit. From its apex in front there descends a delicate and semi-spiral spine, twisted from within, outwards, that in the prepared skull reaches about half way down to the quadrato-jugal bar.

These lacrymal bones are much alike in a great many species of gallinaceous birds, and I found them in all our American grouse much as they exist in these wild chickens of India † now under consideration (See Figs. 2, 3 and 4).

* *Ibid.*, p. 320.

† SHUFELDT, R. W. "Osteology of the N. American Tetraonidæ." Hay-

Fig. 2.

Fig. 3.

Fig. 4.

Fig. 7.

Fig. 2. Right lateral view of the skull of *Gallus bankiva* of India.

Fig. 3. Superior view of same skull shown in figure 2. Mandible removed.

---

den's 12th Annual U. S. Geol. and Geogr. Survey of the Territories. Plates x. and xiii., figs. 71, 73, 88 and 89. Author's ed. publ. separately, entitled "Contributions to the Anatomy of Birds," Washington, Oct., 1882.

Viewed as a whole, the superior aspect of the skull in both cock and hen *Gallus bankiva* is smooth, and presents us for examination a pair of dome-like eminences posterior to the orbits and formed by the frontal bones, while the interorbital area is broad and quite flat. Longitudinally in the median line, from parietal region to the shallow excavation between the lacrymals there runs a faintly-marked groove, most evident in the male bird, which is less pronounced in front than it is posteriorly. In this groove in the male, and beneath the site of the comb, there is to be found a fairly well marked elevation, of which there is not a trace in the hen (Fig. 3). Then again, a pair of inconspicuous and elongated elevations, one occurring on either side of the median furrow, are to be observed immediately in front of the transverse fronto-parietal depression, in which elevations the bone of the cranial vault appears to be thinner, as may be seen by holding the skull up to the light. The parietal region of this superior aspect of the cranium is broad, concave from side to side, gently sloping down on either hand to the tympanic apertures, where the squamosal completes the cranial surface.

Between the orbital peripheries the frontal region of the skull in both cock and hen of these fowls is, as I have said, rather broad, more so in the former than it is in the latter sex, but this is only due to the greater

---

Fig. 4. Inferior view of same skull shown in figures 2 and 3. Mandible removed.

Fig. 5. Superior view of mandible from the same specimen (figs. 2 and 4).

Fig. 6. Hyoid arches of the same specimen as in figures 2 to 5, seen from above.

Fig. 7. Posterior view of the skull shown in figures 2, 3 and 4. Mandible removed.

Lettering: *pmx*, premaxillary; *n*, nasal; *l*, lacrymal; *f*, frontal; *p*, parietal; *so*, supra-occipital; *sq*, squamosal; *q*, quadrate; *qj*, quadrato-jugal; *pg*, pterygoid; *j*, jugal; *v*, vomer; *mx*, maxillary; *pl*, palatine; *d*, dentary; *su*, surangular; *a*, angular; *ar*, articular; *p. ap*, posterior angular process; *pf*, sphenotic process; *eo*, exoccipital; *bt*, basi-temporal; 9, foramen for hypoglossol nerve; 8, foramen for glossopharyngeal and vagus nerves; *oc*, occipital condyle; *ic*, foramen for internal carotid. All the figures illustrating the present paper are life size, and all drawn by the author from the bones of the skeleton of the *male* bird sent from India by Dr. Burke.

size of the skull in the male, a matter we will deal with further on by presenting tables of measurements of these and other parts. It is this region that mounds up and exhibits those extraordinary perforations in the domestic variety known as the Polish Fowl, and which supports those curious bony protuberances in the Horned Fowl, an other variety, the product of man's selective development.*

A great authority says, in alluding to such monstrosities as compared with the characteristics of normal skulls in his review of this part of the avian skeleton in general, that "the spherical bony cyst above the fore-part of the cranium in a variety of common fowl be omitted, though this, like the stunted mandibles of some varieties of pigeon, may rather rank among the phenomena of pathology."†

Turning now to the lateral aspect of our skull of *Gallus bankiva* (Fig. 2), I find that I have above already sufficiently dwelt upon the premaxillary, nasal, and lacrymal elements, and we now have brought fully into view the unusually delicate, zygoma of this fowl, connecting the quadrate with the nasal bones. By the aid of a lens the fine sutural traces upon it showing the landmarks among the quadrato-jugal, jugal, and maxillary divisions, can yet be made out in the skulls of these adult birds.

The peripheral margin of the orbit is seen to be almost a sub-circular arc, as it sweeps from the lacrymal bone to the extremity of the sphenotic process, while its edge is found to be finely serrated for its posterior moiety. Large and capacious, the external osseous ear-conch is of an elliptical outline, permitting a plain view of its base, where exist those several small perforations which lead to the middle or internal ear, as well as the larger Eustachian opening, situated anterior to them. Above and in front of this aural aperture, we are to observe the two lateral processes,

---

* DARWIN, CH. "Animals and Plants under Domestication," Vol. I., p. 320, Fig, 36.

† OWEN, SIR RICHARD. "Comparative Anatomy and Physiology of Vertebrates," Vol. II., p. 65, London, 1866. In this connection see, also, Tegetmeier, P. Z. S , Nov., 1856; and I. GEOFFREY ST. HILAIRE, "Hist. Gen. des Anomalies." Tom. I., p. 287; also, M. C. DARESTE, "Recherches sur les Conditions de la Vie," etc., Lille, 1863, p. 36.

the sphenotic above and the squamosal below, so characteristic of all skulls of Gallinaceous birds. Here in the male jungle fowl, the sphenotic process is somewhat the longer of the two, is compressed from side to side, and within, continuous with the ali-sphenoidal surface of the cranium by means of an ali-osseous extension. Its tip is free, while I find in the female specimen it is completely fused with the end of the squamosal apophysis, thus including a *temporal foramen* between them.

According to Parker this latter state is rather to be regarded as the normal or more constant condition. The squamosal process is here a very thin lamina of bone, laterally compressed, and as in the case of the sphenotic or post-frontal one, directed downwards and forwards. Passing next into the cavity of the orbit, we find the optic foramen large and single, merging, as it does, with the corresponding opening of the opposite side. This is also found to be the case with the foraminal aperture for the first pair of nerves; vacuities may exist, however, on the posterior cranial wall to the outer side of the latter, as they here do in the skull of my female specimen. Beyond these openings the *interorbital septum* is represented by a thin plate of bone, pierced near its centre in the cock's skull by a considerable fenestra, of an irregular outline, while this plate in the hen exhibits only an unbroken surface, as we find it in most North American *Tetraonidæ.*

*Pars plana* is found to be entirely in membrano-cartilage, unossified in the adult; while the mesethmoid rises as a thickened pillar, to spread out above, as usual, as an abutment for the overlying frontals and nasals above. Posteriorly the orbital wall is smooth and gently arched throughout, being concaved in continuation with the concavity of the vault above, which is furnished by the frontal bone. Darwin, when he came to compare the structures to be examined as the base of the skull in the various species of domestic fowls, was forced to remark that "the bones at the base, from the occipital foramen to the anterior end (including the quadrates and pterygoids), are absolutely identical in *shape* in all the skulls. So is the lower jaw."* And, indeed, I fully believe this to hold true with specimens of the wild *Gallus bankiva*; and so well known now are these several structures that it will be but necessary to touch upon them lightly in the present connection, the less imperative is it, too, as I have taken no little pains in my illustrative exhibition of them in Fig. 4 of this article. One thing it will be well to record, however, and we are to note that each and every one of these parts are conspicuous for their *slenderness* as compared with the corresponding structures as we find them in the skulls of most common chickens of barn-yard breeding, wherein such bones as the quadrates, pterygoids and palatines seem to be more heavily fashioned. *Gallus bankiva* has an extremely delicate pair of *maxillo-palatines*, performed in osseous tissue, and separated by quite an interval in the middle space.

Either *palatine* is noted for the slender "maxillary process" which it sends forward to its usual articulation with the bony structures beneath the superior osseous mandible; and those processes are well separated mesially, as are the inner margins of the internal lamina of the palatines along the nether surface of the rostrum of the sphenoid (Fig. 4).

Posteriorly, the "pterygoidal process" of a palatine, turns outwards and articulates in a socket, designed for its reception, in the head of a corresponding pterygoid. As in all true gallinaceous species this jungle fowl has the posterior external angles of the palatines most completely rounded away.

A *vomer*, of the most delicate construction possible, is found to be freely supported upon the tips of the forward projecting "ascending processes" of the palatines, where they nearly meet beneath the apex of the sphenoidal rostrum. This diminutive vomer is forked behind, and as sharp as a needle in front; and I find it better developed in the skull of the male than I do in the skull of the

* "Animals and Plants under Domestication," Vol. I., p. 315. Amer. Edition.

female, whereas I have yet to find such a bone either in our domestic or wild turkey.* And, further, I am almost compelled to believe that it is just possible that this minute element of the basal structures of the skull does not invariably ossify in all specimens of domestic fowls; at any rate it may not do so until they are well advanced in years. Often I have examined chickens of several summers' growth wherein it yet appeared to be in membrane. Professor Huxley found one of no inconsiderable dimensions in the skull of the common fowl, of which he presents us with the figure;† and the same in truth may be said of the admirable illustration given us by Parker,‡ also of a common domestic chicken.

Both *quadrates* and *pterygoids* in *Gallus bankiva* are apparently pneumatic bones, the former possessing the usual pattern as seen generally in the *Gallinæ*, with two mandibular and two mastoidal articular facets; with a blunt-pointed orbital process which is somewhat abruptly bent backwards near its middle, well below which angle of bending we find the semi-globular facet for the quadratal end of the corresponding pterygoid. This latter bone has a shaft much compressed from before, backward, twisted upon itself, and terminating anteriorly in a club-shaped head, so fashioned as to present an elliptical facet for articulation with a similar surface at the side of the rostrum, and more anteriorly a cupped depression to admit the out-turned pterygoidal end of the corresponding palatine. These bones are shown *in situ* in Figs. 2 and 4.

A very meagre lip of bone juts forward as the mesial anterior process of the basitemporal to underlap the entrances to the Eustachian tubes in front; while posterior to this site the basitemporal area itself is broad from side

* For a discussion of this last point see my paper published in a former number of THE JOURNAL, entitled "A Critical Comparison of a Series of Skulls of the Wild and Domesticated Turkeys (*M. g. Mexicanus*, and *M. g. domestica*), Vol. VIII., No. 3, Art. XX., pp. 207-222, July, 1887.

† HUXLEY, TH. H. "The Anatomy of Vertebrated Animals," p. 242, Fig. 82, N. Y., 1872.

‡ PARKER, W. K. Art. "*Birds*," *Encycl. Brit.*, 9th Ed., Vol. III., p. 710, Fig. 21, *v*.

to side, and much convexed in the anterio-posterior direction. Just within the otic margin, on either side, we find the usual group of three foramina, for the passage of nerves and vessels (Fig. 4, *i.c.*, 8 and 9). Quite an excavation exists in front of the single, small and superiorly notched occipital condyle, above which is seen the rather large, subcircular foramen magnum.

The occipital area on the posterior aspect of the cranium (Fig. 7) is faintly bounded by a raised, subcordate ridge, and the rather well-marked supra-occipital prominence is unpierced by any formina in either of my specimens. Referring to the outline assumed by the *foramen magnum* in fowls, Darwin once more points out for us some of its varying features when he says that "the most remarkable character is the shape of the occipital foramen; in *G. bankiva* the breadth in a horizontal line exceeds the height in a vertical line, and the outline is nearly circular; whereas in Cochins the outline is subtriangular, and the vertical line exceeds the horizontal line in length. This same form likewise occurs in the Black Bantam above referred to, and an approach to it may be seen in some Dorkings, and in a slight degree in certain other breeds." *

Nothing worthy of special note, beyond what we already know, characterizes the small, intrinsic ossicles of the otic organ; and in the eye we find from thirteen to sixten well-ossified "sclerotic plates," of which the anterior ones, as they are arranged in the circumpupilar circlet, are not so wide nor so deep as the more posterior ones; indeed, as they pass round, overlapping each other in their course, they gradually increase in these dimensions, till we arrive at the hindermost one of all, which is usually the biggest one.

Directing our attention next to the *mandible* of *Gallus bankiva*, we find its form accurately portrayed in Figures 2 and 5, and it is seen to be a V-shaped bone, with a shallow symphysis, and all its many separate elements here thoroughly fused in the adult Jungle Fowl. Lacking entirely a ramal vacuity, it also developes the badge of its tribe in the backward projecting posterior articular process (*p. ap*), so prominent in all gallinaceous species.

* "Animals and Plants Under Domestication." Vol. I., pp. 315, 316.

More delicately constructed, yet agreeing in all essential particulars, the "hyoid arches" of this cock are much as we find them in the ruler of the dung-hill, our modern rooster, as may be seen by inspecting my drawing of them in Fig. 6 of the present paper; and Parker, through his many clear descriptions, and more than instructive figures, has so impressed the several parts, and the development of these "arches" upon the minds of all who have ever looked into such subjects, that further description here, beyond my drawing, would indeed be superfluous.

In the hen of *G. bankiva*, they agree with the male bird, except as I have already indicated, in the point of size, being proportionately smaller.

Upon comparing the skulls of wild and domesticated turkeys, in my paper, to which I have already alluded to above, I was made to say that "when we come to simply superficially compare the skull of one of these wild turkeys with the skull of one of the domesticated ones, we appreciate that same difference which we find upon a similar comparison to distinguish the skull of a cock *G. bankiva* and any of the typically domesticated fowls. It seems to consist in a lightness, a pneumasticity, accompanied by a certain sharpness of the details of the skull, an angularity, if we may so express it, in the case of the wild bird, as contrasted with an evident thickness and density of the bone, together with a general mellowing down of its principal edges, producing a certain lack of sharpness in the case of the domesticated one." From a survey of the since-acquired material, I am prepared to say that these remarks and conclusions are quite as true to-day as the day they were written, over a year ago. Moreover, I predict that if complete measurements of the brain-case and the *size* of the brain are ever made for a series of adult males of the wild *Gallus bankiva*, and compared with similar data obtained from a like series of domestic fowls of corresponding general proportions, that the average size of the brain in the former will be greater than the average size of the brain in the latter, all else being equal. From this I mean to say that I firmly believe that our domesticated varieties of fowls have deteriorated mentally since the days they were first domesticated by man ; and now, in this particular, the wild species are their peers.

Comparing next the skulls of our male and female *G. bankiva* by measurement, and using centimetres and their fractions as our scale, we note some of the following differences :

| Distance between certain points on the skull. | Male. | Female. |
|---|---|---|
| Greatest median longitudinal length........................ | 6.1 | 5.5 |
| Greatest width, from tip of one sphenotic process to the other on the opposite side.................................. | 2.6 | 2.4 |
| Greatest height, vertex to mid-point basi-temporal area...... | 2.3 | 2.1 |
| Length of side of mandible............................... | 4.9 | 4.4 |
| Distance between apices of the posterior articular processes of mandibles.......................................... | 2.7 | 2.2 |
| Height of foramen magnum.............................. | 0.5 | 0.5 |
| Distance between the quadrates........................ | 1.45 | 1.3 |

*Of the Remainder of the Skeleton.*—Both the cock and the hen of my specimens of *G. bankiva* possess fourteen vertebræ in the cervical region of the spinal column, before we come to one that bears a pair of freely articulated ribs, be these latter great or small. This cannot agree with what Darwin found in his skeletons of the wild *G. bankiva*, but this observer noted that as he passed to some of the domestic varieties or species of fowls, that "in two Games, in two pencilled Hamburghs, and in a Polish, the fourteenth vertebra, bore ribs, which, though small, were perfectly developed with a double articulation." * In the specimens of the Jungle Fowl before me, the first fourteen vertebræ of the column are quite alike in both sexes, except in point of size, those of the male being proportionately the larger.

Choosing these latter then for a few descriptive remarks, we are to note that in the case of the *atlas*, the upper part of its occipital cup is roundly notched out in order to admit the "odontoid process" of the axis. This latter verbra possesses a tuberous neural spine, and below, a con-

*Animals and Plants under Domestication. Vol. 1, p. 322.

spicous, sharp, hypophysis. In the third, fourth and fifth segments this last-named process is very prominent, being long and sharp ; in the fifth vertebra, however, it is less so than in the first two mentioned. Parapophyses commence on the third vertebra, and are in mid series long and spiculiform. In the fourth vertebra the pre- and post-zygapophyses are joined on either side by a lamina of bone, which, in each case is perforated by a small foramen. From the fifth to the eleventh vertebræ inclusive, we find the hæmal processes modified in the usual manner, so as to form a canal for the passage of the two carotids. Laterally, the vertebral canal passes on either side, from third to the thirteenth vertebra inclusive.

A very handsome lamelliform hypophysis, directed forwards, occupies a median position upon the nether aspect of the twelfth and thirteenth vertebræ, a character also of the fourteenth and fifteenth segments, where, however, they are considerably smaller. A well-developed, knob-like neural spine is upon the usual site in the fourteenth vertebra, situated far back, between the post-zygapophyses.

Passing next to the fifteenth vertebra of the column, we find that it has a strong, quadrate neural spine, and quite prominent and thick diapophyses. From below these latter are suspended the first pair of free ribs. These ribs, in my male specimen, are each but a centimetre long, while in the hen they lack but a millimetre of being two centimetres long. In both, the tubercula and capitula are well developed, though in neither are there present the uncinate processes. As I have already stated above, this vertebra also has a median hæmal spine of no great size ; it has more than this, as we see a smaller spine, one projecting from beneath the centrum, upon either side of the median apophysis. Next follow in the spinal column of *G. bankiva*, *four* true dorsal vertebræ which solidly fuse together, forming a single bone, which I have drawn upon lateral view in Fig. 20 of this paper. Its neural spine consists of a continuous, lofty and quadrilateral osseous plate, finished off along upon its superior margin by a bony, raised rim. Its diapophyses are wide-spreading and thoroughly joined together

Fig. 8.

Fig. 9.

Fig. 10.

Fig. 11.

Fig. 12.

Fig. 8. Left lateral view of the sternum of *Gallus bankiva*, of India.

Fig. 9. The same bone as shown in figure 8, seen directly from beneath.

Fig. 10. The *os furcula* of *Gallus bankiva* viewed directly from behind.

Fig. 11. The right coracoid of the same specimen seen from in front.

Fig. 12. The right scapula, of the same bird, its dorsal aspect.

at their outer extremities by linking metapophyses. Three neural foramina pierce its sides, while the fused centra are much compressed laterally. There are also four complete facets for the heads of ribs, and four others for the tubercula of the same, at the ends of the transverse processes. The neural canal, passing through this complex bone, is nearly cylindrical in form, and of but moderate calibre. Longitudinally, the median crest below the centra is very sharp along its lower edge, and throws down a fused hypapophysis of a form shown in Figure 20. Other gallinaceous fowls have this bone of somewhat different form,* and it is quite characteristic of many species of the order.

Now the first pair of ribs that articulate at the anterior end of this dorsal bone of the spinal column, are freely suspended, and support a large "epipleural appendage," in each case ; and this latter, as in all these appendages or *uncinate* processes, they are loosely articulated to the borders of the ribs behind.

No marked difference distinguishes this second pair of ribs of the vertebral column in the male from those in the female, and I believe it will never be found in *G. bankiva*, that they ever connect with the sternum by costal ribs, or hæmapophyses, as these latter are sometimes more properly designated. Following this first pair of ribs that articulate with the fused dorsal bone of the column, we always find in this species, three other pairs of fully developed and true ribs that have uncinate processes, and connect with the sternum by the intervention of hæmapophyses. Figure 19, of the the present paper, presents an anterior view of the next vertebra of the spinal column, which in *G. bankiva* is freely inserted in the adult fowl between the coössified dorsal bone and the anterior one of the pelvic sacrum. Its ribs, too, connect with the sternum by costal ribs, which latter are long, and have latterally compressed posterior extremities. Uncinate processes may or may not occur upon this pair of vertebral ribs ; they are present and anchylosed in my male specimen, and altogether absent in the hen. Thus we have *four* pairs of ribs that connect by

---

*See the writer's "Contributions to the Anatomy of Birds," p. 704, Pl. VI., Fig. 55, for the bone in *Centrocereus*. Washington, 1882.

others with the sternum, and I must believe this to be the normal arrangement in the case of the species before us. Darwin has amply shown that it varies widely in many of the domesticated fowls, and from his and my own studies, I am inclined to believe that the time will come when there will appear domesticated races of fowls in which all the vertebræ in the adult, from atlas to pelvis inclusive, will remain free segments, and coössification in the dorsal region not occur. *Gallus bankiva* also normally possesses "sacral ribs," which spring from the leading fused vertebral of the pelvic sacrum, are long and slender, and without uncinate processes. At their lower ends they articulate with hæmapophyses. Each one of these latter bones has a much-expanded and laterally compressed posterior extremity, while anteriorly its end articulates with the hinder margin of the ultimate hæmapophysis, at a short distance above the costal border of the sternum of the corresponding side. Briefly recapitulating then, we find that *G. bankiva* normally possesses *seven* pairs of ribs; the first two pair fail to connect with the sternum, which is the case with four pairs that succeed them; finally there is a seventh, or sacral pair, which articulate below with what may be called a pair of "floating ribs," not using, however, this latter term quite in its anthropotomical or even crocodilian sense.

Perhaps of all the larger bones of the axial skeleton, the *pelvis* has retained its primitive form more than any other among the many domesticated breeds as compared with that bone in the original stock of them all, the *G. bankiva* at my hand. I felt that my work upon this part of the skeleton was more than half accomplished when I completed the drawings presented in Figs. 13, 14 and 15, and yet how little the last-named one differs from Parker's figure of the pelvis in the common barn-yard fowl.*

To be sure Darwin found that the anterior margin of the ilium varied from a rounded to a truncate outline; that the extremity of the pubic bones were "gradually enlarged

---

*See Fig. 34, *Encycl. Brit.*, 9th Ed., p. 722, and numerous copies elsewhere.

in Cochins, and in a lesser degree in some other breeds; and abruptly enlarged in Bantams." *

Fig. 13.

Fig. 14.

Fig. 15.

Fig. 13. The pelvis of *Gallus bankiva* of India, its dorsal aspect.

Fig. 14. Same bone seen upon ventral view.

Fig. 15. Right lateral view of same bone shown in Figures 13 and 14.

---

* *Ibid*, p. 324.

Careful count assures me that there are fifteen vertebræ included in the consolidated pelvic sacrum, of which the first four throw out their diapophyses to abut against the nether surface of the ilium upon either side. In both of my specimens the *propubis* is very large, while the postpusisis long and slender, scarcely touching the lower margin of the ischium for its entire length. It projects about a centimetre beyond the latter bone posteriorly, and shows but a slight tendency to enlarge at this end. The ischiadic foramen is broadly rounded anteriorly, gradually sloping in outline to a point behind ; this is also the form of the opening on the right side in my female specimen, but strange to say, on the left side of the bone there, the aperture is nearly of a circular outline. The inner margins of the post-acetabular portions of the ilia are but placed in close approximation with the corresponding borders of the sacrum, with which latter they do not anchylose, while anteriorly these juxtaposed margins are completely fused together. Strongest among the braces afforded by the transverse processes of the vertebræ of the pelvic sacrum to the ilia on either hand, are the diapophyses of the *first*, *fourth* and *tenth*, and these seem to be somewhat modified to meet this very end (Fig. 14). Gallinaceous birds as a rule all have a capacious pelvic basin, and the jungle fowl before us affords no special exception to it, for the concavity here is both deep and wide, making ample room for the organs and viscera it protects during life.

*Six* vertebræ are to be found in the skeleton of the tail of *Gallus bankiva*, to which is to be added a curiously formed *pygostyle*. My male specimen has all six of these caudal vertebræ free, whereas in the skeleton of the female, the anterior one has fused with the ultimate urosacral. We must believe that Darwin made a slip in his count, when he reckoned "seven" caudals for this form, for were that so, and he seems to have included the last sacral in his number, he could not but have claimed fourteen for the pelvic sacrum, whereas, as he rightly records, there are fifteen. Strictly speaking, *G. bankiva* has *four* dorso-lumbar vertebræ, *five* sacrals, and *six* urosacrals in the sacrum of its pelvis. Even this is at variance with Huxley's count, for these segments in the sacrum of a young chick of the common barn-yard species, where he makes but *five* urosacrals.* Nor do I believe we can be safely guided in this matter by the "double foramina" for the exit of the spinal nerves, for in the pelvis of the male bird before me, the first of these is found just *anterior* to the transverse process of the last dorso-lumbar, and counting this pair of foramina as number one, we find it followed by *eight* other similar "double foramina" as we proceed towards the urosacrals. Professor Parker's drawing seems to me to miss it just in the other direction, for he gives us in the sacrum of a "young fowl" but *four* sacrals and *seven* urosacrals; this, however, is much better as it makes the *total* count *correct*;† and in doing so sets Darwin's figures aright.

Jungle Cocks, as will be seen from Fig. 15, have the supero-posterior angle of the *pygostyle* drawn out into a long, spine-like process, and this seems to be approached by other gallinaceous species, as, for example, the *Centrocercus* of the Western plains of the United States.‡

Excepting the atlas, axis, ribs, and caudal vertebræ, I find that the parts of the axial skeleton which we have been considering in the present section to be quite thoroughly pneumatic, perhaps some portions of the pelvis being less so than any, while many of the vertebræ are highly so.

Before proceeding to the consideration of the shoulder-girdle and sternum, I will add here a few comparative measurements of the pelves of the male and female *G. bankiva*, employing as above the metric scale.

---

* The Anatomy of Vertebrated Animals, p. 238, Fig. 80, *c*.

† Art. "Birds," 9th Ed. *Encycl. Brit.*, Vol. III., p. 719, Fig. 29.

‡ Shufeldt, R. W. "Contributions to the Anat. of Birds," p. 710, pl. IX., Figs. 65 and 66. Washington, 1882.

| Distances between certain points on the pelvis. | Male. | Female. |
|---|---|---|
| Greatest longitudinal median length... .................. | 6.8 | 5.7 |
| Greatest absolute length.............................. | 9.2 | 7.7 |
| Tip of one propubis to tip of other.................... | 3.7 | 3.2 |
| Greatest absolute width............................... | 4.3 | 3.8 |
| Greatest absolute heighth............................. | 2.8 | 2.3 |
| Least width................ ........................ | 2.3 | 1.9 |
| Length of post pubis...................... ..... | 5.4 | 4.6 |
| Distance between bases of the acetabula............. | 3.1 | 2.6 |
| Length of pelvic sacrum............................... | 6.3 | 5.3 |
| Greatest width of pelvic sacrum.................... ... | 2.0 | 1.7 |

In making the measurements in the case of the pelvis of the female in this table I was careful not to take into consideration the caudal vertebræ which we found united with the sacrum in this specimen.

*Of the Sternum and Shoulder Girdle :* So well known is the general form of the sternum among typical *Gallinæ*, and in *Gallus* in particular, that it would be more than superfluous labor for me to enter upon a detailed description of the bone in the present connection. My figures faithfully portray its form in the adult male *G. bankiva* (Figs. 8 and 9), and Darwin has told us that in the case of domestic species he found out of twenty-five sternums examined by him, that "three alone were perfectly symmetrical, ten were moderately crooked, and twelve were deformed to an extreme degree."* It is a well known fact that it is a rare thing to come across a *perfectly* symmetrical sternum from a common domestic fowl, whereas it is truly an elegantly fashioned bone, not only in *bankiva*, but in many of its allies as the Grouse and Partridges.† Much of this is due to the graceful sweep of its deep keel, its lofty costal processes, its wide-spreading and delicate xiphoidal limbs; and its handsome manubrium, transversely pierced at its base by a communicating foramen connecting the

* *Ibid*, p. 330.

† For examples of these latter (*i. e.*, the Grouse), see the writer's figures in his "Contrib. to the Anat. of Birds," p. 714, Figs. 81 and 82, and p. 704, Fig. 54, and for the Ptarmigan, in the same work, p. 718, Fig. 91. Washington, 1882.

costal grooves. (See Figs. 8 and 9). In *G. bankiva*, too, the sternum is highly pneumatic, and perforations for the admission of air into its substance are to be found in the little valleys among the facets for the hæmapophyses upon the costal borders; and more extensive ones upon the far anterior aspect of its thoracic surface, or in the median, longitudinal furrow behind these latter.

Not content with simple appearances, Darwin even went further than I have hinted at in the last paragraph, for he made many proportional measurements, among depth of carina, length of bone, etc., etc., for our present subject as compared with the domesticated species; and to those comparisons I will add here the differences in size of the bone in the adult male and female,—data which, for the end he had in view at the time, was not especially called for, and consequently not presented. These measurements I will offer in another "Table," after we have briefly considered the shoulder girdle.

Let any one take the pains to compare the excellent figure of Professor Parker's drawing of the *pectoral arch* chosen from a common barn-yard fowl, and presented us in the ninth edition of the Encyclopædia Britannica, Vol. III., p. 720, with my drawing here given (Fig. 16), of the same parts for *G. bankiva*, and it will not be hard for him to admit that the bones of the wild fowl have a more delicate, graceful, and withal, elegant appearance, than those of the long domesticated species. And in truth so it is. In *G. bankiva*, the limbs of *os furcula* are slender and subcylindrical, more especially so in the hen where this bone is a very delicate structure, while its coracoidal ends are but moderately expanded in either sex. Chiefly, however, is to be noticed its large and sub-triangular hypocleidium, with its salient angles nicely rounded off, and its broader moiety, pendant.

A *coracoid* possesses but a fairly tuberous head, with its summit hooked over mesiad, so that when the arch is articulated *in situ*, it largely shares in forming the "tendinal canal," and allows the corresponding head of the os furcula to rest against it, but not the head of the scapula of the

Fig. 16

Fig. 17.

Fig. 18.

same side. Below, the sternal extremity of the coracoid is moderately expanded, but offers nothing peculiar as to form. The shaft is long, straight and slightly compressed from before, backwards. For the "glenoid cavity," the coracoid offers about two-thirds of the articulatory surface; the scapula furnishing the remainder.

Either *scapula* presents in its head or anterior end the usual ornithic characters common to so many of the *Carinatæ* in general, and to all true *Gallinæ* in particular; for we find its acromial process, when the bones of the arch are articulated *in situ*, extending forwards to meet the superior end of the *os furcula*, and its glenoidal process completing, as usual, the cavity for the head of the humerus. Narrow, long and slightly curved, its blade in the skeleton reaches back to the pelvis or more, and is characterized by having a smooth, rounded outer margin, and a sharp upturned inner one, at least for, in the latter case, its posterior four-fifths. Its distal apex is somewhat dilated. Possibly the scapula may be pneumatic; the coracoid always is in this species, but the *os furcula* never so.

| Distances measured in metric system. | Male. | Female. |
|---|---|---|
| Length of keel in sternum | 7.9 | 6.8 |
| Greatest depth of keel in sternum | 2.9 | 2.6 |
| Total length of sternum | 10.3 | 8.9 |
| Distance between apices of costal processes of sternum | 3.0 | 2 2 |
| Distance between tips of outer pair of xiphoidal processes of sternum | 4.7 | 4.3 |
| Length of os furcula | 5.8 | 5.0 |
| Length of coracoid | 5.0 | 4·1 |
| Length of scapula | 6.6 | 5.8 |

*Of the Appendicular Skeleton.—The Pectoral Limb.*—As compared with the rest of the skeleton, the limb-bones of domestic species of *Gallus* have in the various modifications that have taken place in time in them, since deviation from the *bankiva* stock first commenced, been of a far less profound character; and that master observer, Darwin, says upon this point, that, "I have carefully compared each separate bone of the leg and wing, relatively to the same bones in the wild Bankiva, in the following breeds, which I thought were the most likely to differ; namely, in Cochin, Dorking, Spanish, Polish, Burmese Bantam, Frizzled Indian, and black-boned Silk Fowls; and it was truly surprising to see how absolutely every process, articulation and pore agreed, though the bones differed greatly in size. The agreement is far more absolute than in other parts of the skeleton. In stating this, I do not refer to the relative thickness and length of the several bones; for the tarsi varied considerably in both these respects. But the other limb-bones varied little, even in relative length." *

As to the extent they may vary in length and general size, I would again invite the reader's attention to Professor Parker's drawing of the limb-bones of a common barn-yard fowl, which may be compared with those I present with this paper (Figs. 17 and 18), as accurate illustrations of the corresponding parts in *G. bankiva.*†

* Animals and Plants under Domestication. Vol. I., p. 325. N. Y., 1868.

† Art. "Birds," *Encycl. Brit.*, 9th Ed., Vol. III., p. 721, Fig. 33. Similar comparisons may also be made for the several bones of the pelvic limb, taking

Presenting the usual sigmoid curves in the continuity of its smooth and somewhat compressed shaft the *humerus* of our jungle cock is a thoroughly pneumatic bone, the fossa harboring the foramen being well overarched by the ulnar tuberosity.

Between this latter and the large ellipsoidal head, there exists a rather deep and circumscribed pit or valley, while another and shallower excavation is to be found just beyond the humeral head on the anconal aspect of the shaft. The radial crest is moderately prominent, while at the distal extremity of the bone both radial and oblique tubercles are more than usually conspicuous.

Along the bowed and heavy shaft of *ulna* we note peculiar markings denoting the sites where the butts of the secondary quills are inserted; these, however, are not elevated into papillæ as in some avian types; and this bone is to some degree, especially its proximal moiety, laterally compressed, and withal thoroughly non-pneumatic, as are the remaining skeletal parts of this limb.

*Radius* is straighter than ulna, being but slightly curved downwards in the vertical plane; while its shaft, too, shows some lateral flattening, but in its case, along the distal half of the bone, the very reverse of its companion in the antibrachium. Thus formed, it is evident that a considerable "interosseous space" must exist in the skeleton between these long bones of the forearm, which is really the case (Fig. 17). *Carpus* offers us the usual *radiale* and *ulnare* segments fashioned almost identically as we find them among the *Gallinæ* generally, and having precisely the same articulatory relationships. Passing to the skeleton of *manus*, we are at first principally struck with what might be termed the comparative strength of the parts. There is a moderate approach towards massiveness in the pinion-bones of any fowl of the genus *Gallus*, and the wild species offers no exception. Others have noted the relative shortening of the manus and antibrach-

---

Prof. Parker's drawings from the same article (Figs. 35, 36 and 37), and con trasting them with my figures here given for the same bones in *G. bankiva*. (Figs. 21 to 30 inclusive.)

ium in the gallinaceous types,* while still others render a description without special comment.†

Notable among the points to observe in our present subject, the skeleton of the hand in *G. bankiva*, are, the small claw on the large free phalanx of pollex digit; the overlapping process on the postero-proximal aspect of the shaft of the second metacarpal, which rests by its apex upon the juxtaposed part of the shaft of the third metacarpal; this feature is characteristic of all true *Gallinæ*;‡ the broad, non-perforated blade of the proximal phalanx of the index digit; and finally the comparatively diminutive size of the phalanx of the last metacarpal.

A glance at my drawing in Fig. 17 will be sufficient to convince one that the possessor of a wing such as its skeleton there suggests, could be nothing less than a fowl of no little powers of flight, and so, I believe, is the case in the wild *G. bankiva;* yet we often meet with domestic species with equally good wings, that prove to be among the most indifferent or even helpless of flyers. And this is a very interesting question, and so far as my opinion goes, I am inclined to think that the muscular system is the one most at fault, and from the long-continued *habit* of *not* flying, the muscles have largely lost, by this time, both power and education in this particular. May-be at the end of the next chapter in the history of these domesticated galline races, the bones of the pectoral limb will show decided steps in the direction of permanent atrophy—say 6,000 years from now. Very likely in some barn-yard species, the weight, as in the case of the Cochins, has something to do with the matter, inducing an *habitual disinclination* for flight. Some twenty-five or more years ago, the writer owned a flock of pure breed game fowls, the hens being all of a plumage and very wild in habit. These

---

* CHAUVEAU, A. The Comparative Anatomy of the Domesticated Animals p. 117, New York, 1884. (Fleming Edition.)

† M'FADYEAN, J. The Comparative Anatomy of the Domesticated Animals. Pt. I., Osteology, p. 166, N. Y. 1888.

‡ SHUFELDT, R. W. "Osteology of the N. American Tetraonidæ." Dept. of the Int. U. S. Geol. and Geogr. Surv. of the Terr. Hayden's 12th Ann., p. 706, Fig. 58, Washington, 1881.

chickens when alarmed thought little of springing from the ground together, and taking a flight of some five or six hundred yards ; the character of the flight being much as we see among quails. And if I may be allowed here a still greater digression, I may add that I once saw a number, some dozen or more, tame turkeys fly together over a half mile, and light upon the very tops of some tall hickory trees on the skirts of a forest. They had been suddenly alarmed by a firearm's discharge ; and yet these birds had been notorious for several years as being more than clumsy flyers, a fact that had been noted as they went to roost at night. These, however, are simply cases wherein sudden fright seems to stimulate the long latent power, which otherwise the past ages of disuse and inheritance are slowly but permanently abrogating.

*The Pelvic Limb.*—Much that I have hinted at in the foregoing paragraphs with respect to the skeleton of the arm, applies with equal force to the leg ; though in domestic chickens there is every reason to believe that this latter part of the skeleton will tend in time to rather become stronger than otherwise, from greater use.

The wild cock *G. bankiva* has a *femur* of a form and size as we have represented it in two views in Figures 21 and 22. It will be seen that the trochanterian crest is very prominent, and inclined to arch over the summit of the bone. Some semblance of a neck supports the "caput femoris," which latter is but feebly marked, at the usual site, by a pitlet for the ligamentum teres. Adown the shaft we note the usual muscular lines, and this part of the femur is much bowed to the front, and for its middle third, at least, is cylindrical in form. The external condyle is the larger, and situated the lower on the shaft, being cleft as usual posteriorly, to admit in articulation the head of the fibula. The femur, as in the case with all the other bones of the pelvic limb of this bird, is non-pneumatic.

A sizable, transversely elongated *patella* is present (Fig. 29).

*Tibio-tarsus* has its cnemial crest but slightly elevated above its summit, while the pro- and ectocnemial processes are low, twisted to the outer aspect, and soon merge into the shaft in front. This latter is nearly straight, sub-elliptical upon mid-section, and longitudinally furrowed for its lower third, anteriorly. In the male, the fibular ridge is nearly two centimetres in length, and occupies the greater part of the upper third of the shaft, on its outer aspect. Distally, and in front, we find the usual little osseous bridge for tendinal confinement, just above the condyles. Of these latter, the outer is the thicker and most rounded, while posteriorly the surfaces of both merge together.

In the *fibula*, the head is large and produced backwards; the bone never anchyloses with the tibio-tarsus in this species, and after passing the fibular ridge dwindles to a mere thread, being produced to a point something over a centimetre above the external condyle of the main bone of the leg (Figs. 27, 28 and 30).

Ever full of interest to the ornithotomist, the *tarso-metatarsus* in *G. bankiva* is the more especially so, on account of the conical osseous calcar which is firmly anchylosed to the roughened longitudinal line and to the shaft at the lower third of its length.

To its base and mesial aspect this calcar or bony spur-core is worn absolutely smooth and shiny by the constant chafing of the ossified neighboring tendons which bear against it during the life of the bird. The "hypotarsus" of this bone is roughly cubical in form; has one complete, perforating tendinal passage to its inner aspect, and posterior to which the longitudinal margin, mesiad, is thickened and terminated below as a sharpened process (Fig. 23). For the rest, to its outer side, two faint tendinal grooves traverse it lengthwise. Anteriorly, the tarso-metal tarsus is guttered out for the full length of its squarish shaft—faintly so for its distal moiety, conspicuously so, proximad—the latter gradually shallowing as we pass from above, downwards (Fig. 24).

At the lower end of the bone we find the usual arterial foramen perforating it; and trochleæ here are large and prominent (Fig. 25). A distinct facet is seen above the inner one, intended for the articulation of the rather large "accessory metatarsal" of this fowl.

Coming next to the skeleton of pes in this wild *G. bankiva*, we find the arrangement of the osseous phalanges

| Comparative lengths of bone in pectoral and pelvic limbs, given in the metric system. | Male. | Female. |
|---|---|---|
| Length of humerus | 6.6 | 5.6 |
| Length of ulna | 6.5 | 5.4 |
| Length of radius | 5.9 | 4.8 |
| Length of metacarpus | 3.5 | 3.0 |
| Length of pollex digit | 1.4 | 1.3 |
| Length of pinion including the phalanges of digit | 5.7 | 5.0 |
| Length of femur | 7.4 | 6.2 |
| Length of tibio-tarsus | 10.4 | 8.6 |
| Length of fibula | 7.9 | 6.0 |
| Length of tarso-metatarsus | 7.45 | 5.7 |
| Length of basal joint of hallux | 1.2 | 1.0 |
| Length of basal joint of mid-anterior toe | 1.5 | 1.4 |
| Total length of mid toe | 5.0 | 4.3 |

of the several digits to be upon the most normal plan of 2, 3, 4 and 5, reckoning from hallux to fourth toe inclusive, while the ungual joints are in each case strong and powerfully curved, and taken as a whole, although offering nothing of particular note, the skeletal pedal structure of *Gallus bankiva* unmistakably denotes the predominance of its rasorial habits over any arboreal proclivities it may possess. So much for the skeleton of the species of *Gallus*, from which during the long ages gone by, all of our domestic species have been most undoubtedly derived, and that by the action of laws and processes of which we to-day have some good strong inkling, but are by no means thoroughly informed about.

## Figures and Tables

**Fig. 1. f1:**
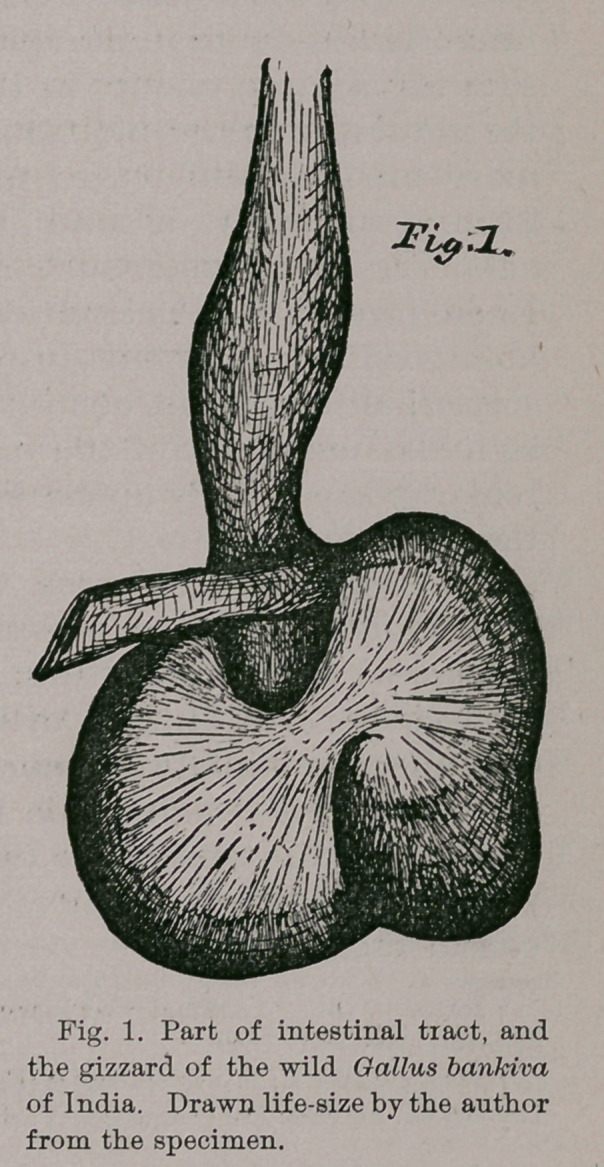


**Fig. 2. Fig. 3. Fig. 4. Fig. 5. Fig. 6. Fig. 7. f2:**
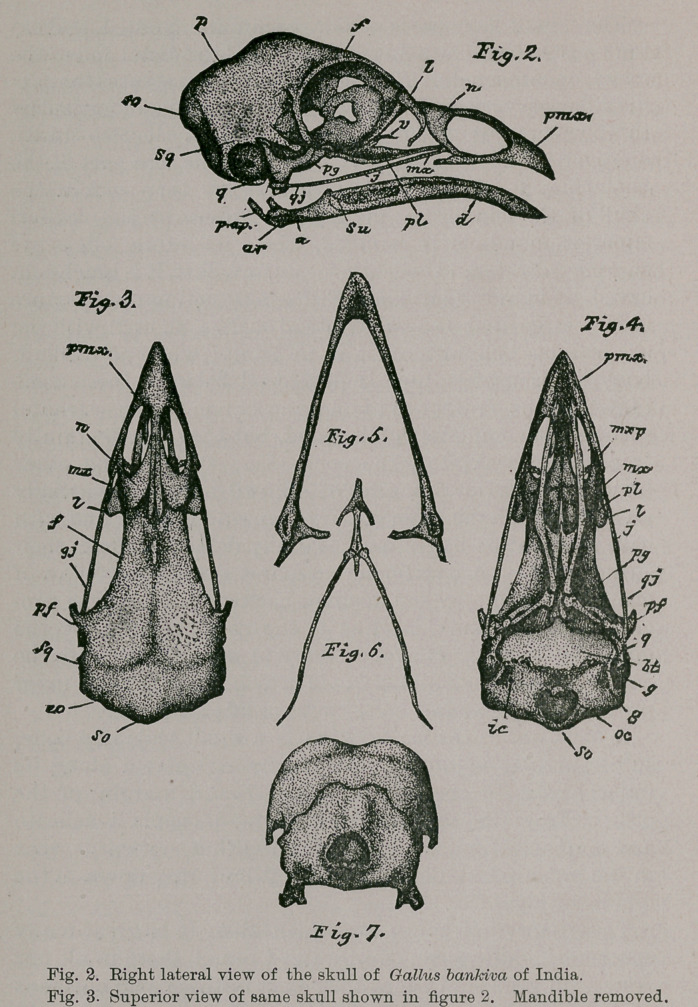


**Fig. 8. Fig. 9. Fig. 10. Fig. 11. Fig. 12. f3:**
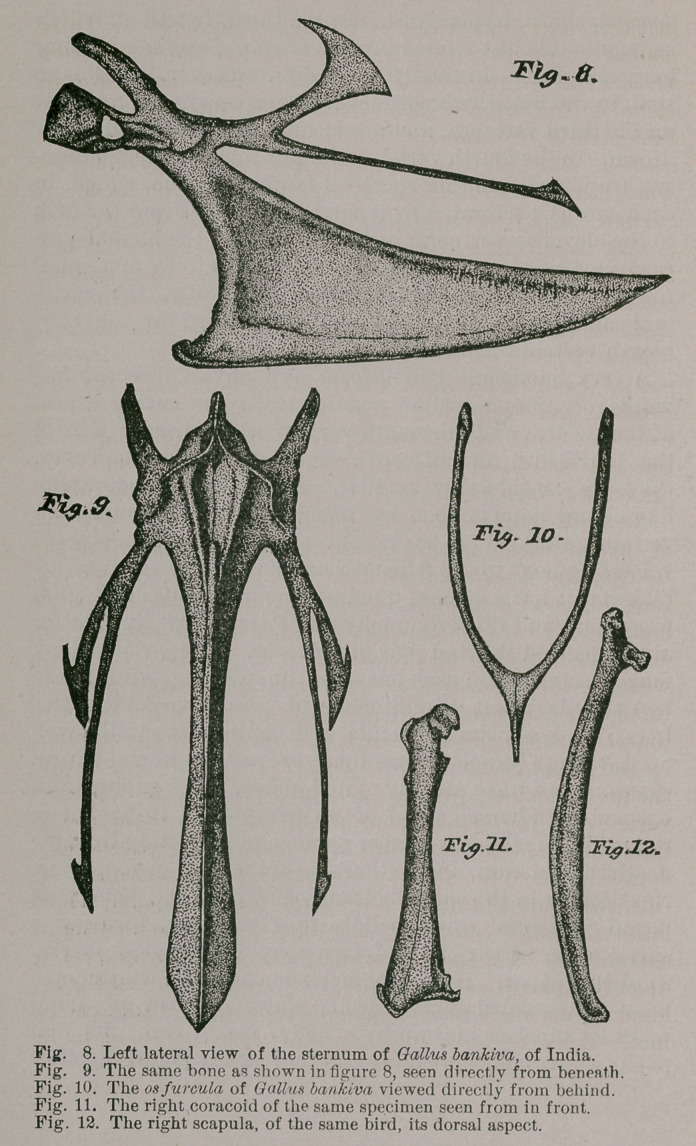


**Fig. 13. Fig. 14. Fig. 15. f4:**
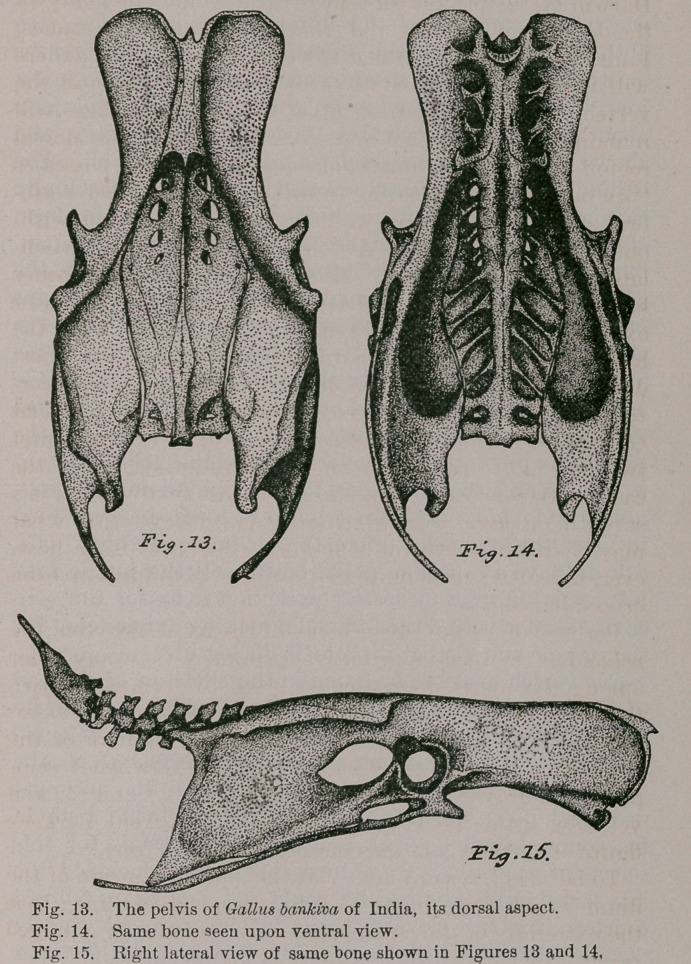


**Fig. 16. Fig. 17. Fig. 18. f5:**
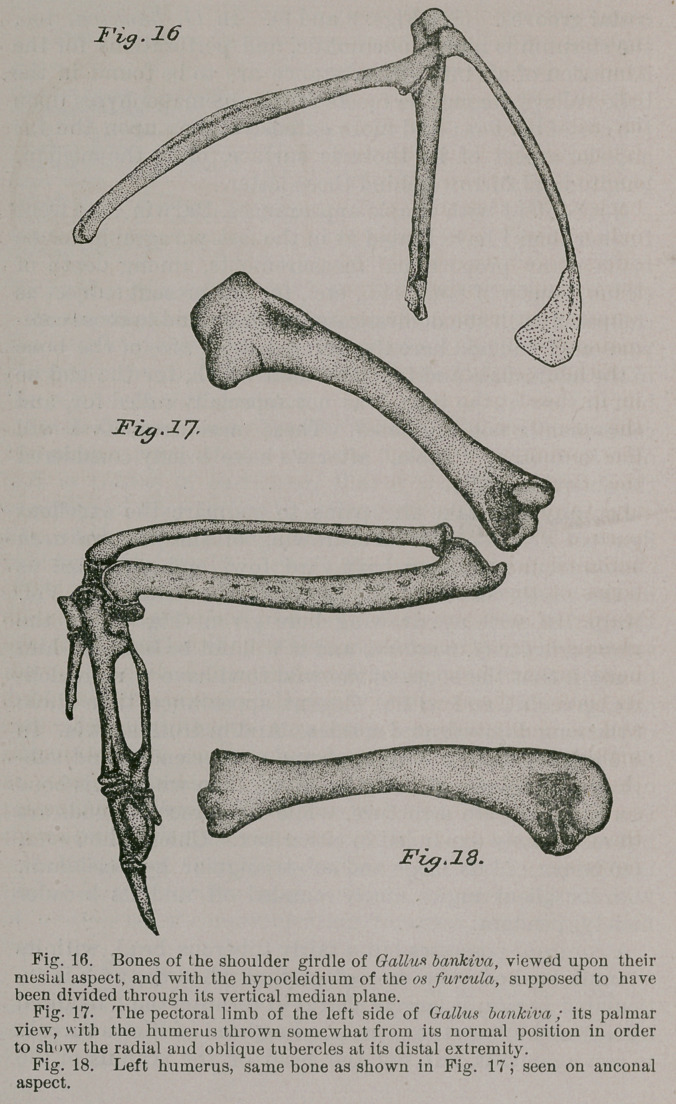


**Fig. 19. Fig. 20. f6:**
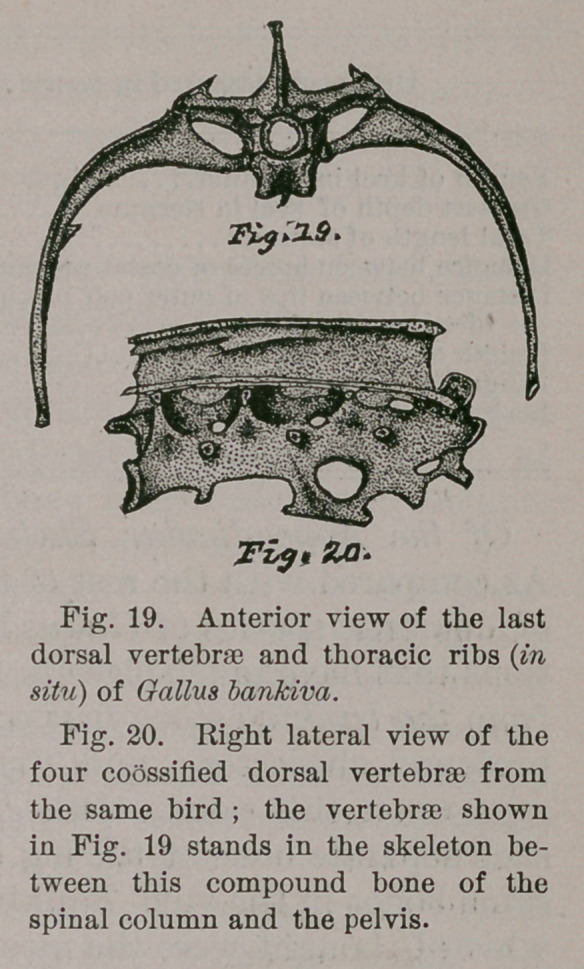


**Fig. 21. Fig. 22. Fig. 23. Fig. 24. Fig. 25. Fig. 26. Fig. 27. Fig. 28. Fig. 29. Fig. 30. f7:**